# Misinformation About Climate Change and Related Environmental Events on Social Media: Protocol for a Scoping Review

**DOI:** 10.2196/59345

**Published:** 2024-10-31

**Authors:** Maryline Vivion, Valérie Trottier, Ève Bouhêlier, Isabelle Goupil-Sormany, Thierno Diallo

**Affiliations:** 1 CHU de Québec-Université Laval Research Center Québec, QC Canada; 2 Department of Social and Preventive Medicine Université Laval Québec, QC Canada; 3 Faculty of Nursing Sciences Université Laval Québec, QC Canada

**Keywords:** misinformation, disinformation, infodemiology, infoveillance, climate change, global warming, greenhouse effect, social media, online social network, environmental health, public support, global challenges, Google, health policy

## Abstract

**Background:**

Climate change and related environmental events represent major global challenges and are often accompanied by the spread of misinformation on social media. According to previous reviews, the dissemination of this misinformation on various social media platforms requires deeper exploration. Moreover, the findings reported applied mainly to the context of the United States, limiting the possibility of extending the results to other settings.

**Objective:**

This study aims to assess the current state of knowledge about misinformation concerning climate change and related environmental events that are circulating on social media. More specifically, we will explore past and current themes, actors, and sources, and the dissemination of this misinformation within the Canadian context.

**Methods:**

This scoping review protocol follows the methodological approach developed by Arksey and O’Malley and advanced by Levac, complemented by the PRISMA-ScR (Preferred Reporting Items for Systematic reviews and Meta-Analyses extension for Scoping Reviews) checklist and the best practice guidance for the development of scoping review protocols. Following the identification of the research questions and assisted by a specialized librarian, we developed search strategies for selected bibliographic databases (MEDLINE, Embase, Web of Science, and GreenFILE) and for gray literature (Google and pertinent databases) searches. Bibliographic and gray literature will be searched to identify relevant publications. In total, 2 members of our team will use the review software Covidence (Veritas Health Innovation) to independently select publications to include in the review. Publications specifically addressing our research questions, peer-reviewed, evidence-based, and published from January 1, 2000, in the full-text version in English or French will be included. Data will be extracted from the included publications to chart, among other items, the years of publication, geographic areas, themes, actors, and sources of the climate change–related misinformation and conclusions reported. Our team will then synthesize the extracted data to articulate the current state of knowledge relating to our research inquiries.

**Results:**

The research questions were identified in January 2024. The search strategies were developed from January to March 2024 for MEDLINE, Embase, and Web of Science and in July 2024 for GreenFILE and gray literature. MEDLINE, Embase, and Web of Science searches were launched on March 26, 2024. The first of 2 rounds of selection of publications identified through these databases was achieved in April 2024.

**Conclusions:**

This protocol will enable us to identify the evolution of themes, actors, and sources of misinformation regarding climate change and related environmental events on social media, including the latest platforms, and to potentially identify a context particular to Canada. As misinformation is known to undermine actions and public support in the fight against climate change, we intend to facilitate the targeting of efforts to combat misinformation related to climate change in an up-to-date and contextualized manner.

**International Registered Report Identifier (IRRID):**

DERR1-10.2196/59345

## Introduction

### Background

Climate change has constituted a major global challenge for several decades [1]. The observed environmental events associated with climate change include global warming, deglaciation, rising sea levels, and increased concentrations of gases leading to the greenhouse effect [1], as well as extreme weather events and natural disasters, such as intense heat waves, forest fires, drought, storms, and floods [2]. Thus, adapting to climate change requires swift modifications of both individual and collective behaviors. However, despite the urgency we face, governmental actions are hindered by, among other factors, disinformation and misinformation [3]. Indeed, disinformation and misinformation create confusion within the population, foster skepticism toward scientific knowledge [4,5] and undermine public support for climate change mitigation policies [6].

Communications regarding climate change are known to be prone to misinformation and disinformation [7]. While misinformation is defined as erroneous information disseminated with no intention to mislead or cause harm, disinformation, which includes fake news and conspiracy theories, is spread with malicious intent [8]. Since the distinction between disinformation and misinformation lies in the intention to deceive, which remains difficult to prove, this article opts to use the term “misinformation” uniformly regardless of whether there is intent to mislead. Terms specific to misinformation related to climate change are also used [8,9]; these include climate skepticism, which refers to views in opposition to the scientific consensus or opposing action to prevent climate change [10], and climate denial, which refers to “misinformation that rejects mainstream climate science” [11].

When unreliable information enters discourse, social media platforms are frequently implicated. Social media are known to provide “fertile ground” for misinformation dissemination [12]. According to studies, misinformation about climate change and related environmental events has circulated through social media, including Twitter (now known as X; X Holdings Corp) [10,13-17], YouTube (Google) [15,18], Facebook (Meta Platforms Inc) [15] and various blogs [11]. By consulting social media, people are thus, inadvertently or through research, exposed to misinformation related to climate change [7,10,11,19]. In addition, the structural, interactive, and social features of social media facilitate and accelerate public polarization [9,11,12] and may reinforce controversy [19,20] surrounding climate change issues. Numerous studies on misinformation about climate change and related environmental events circulating on social media have been conducted [9,11,15,17,19,21,22]. These studies have explored types of climate change denial [22], actors who have propagated misinformation [21], sources of misinformation [9,11,15,21], climate change perceptions [17], or related political ideologies [19].

Several knowledge gaps were identified by previous reviews [9,21]. A “comprehensive review of the academic literature on social media communication on climate change” was published by Pearce et al [21] in 2018. This review highlighted the fact that studies exploring climate change-related communication on newer social media platforms such as Instagram (Meta Platforms Inc) and LinkedIn (Microsoft Corp) should be conducted. An overview was published by Treen et al [9] in 2020, discussing “online misinformation and how it relates to climate change.” This publication identified a lack of knowledge regarding the dissemination of climate change misinformation on social media, calling for future study of this phenomenon. Conducting a more recent review will allow for the highlighting of changes over time in trends tied to the dissemination on social media of misinformation about climate change and related environmental events.

Also, studies on misinformation about climate change and related environmental events circulating on social media reference mainly United States of America contexts [9], which limits the possibility of extending the results to other settings, such as the Canadian context. In fact, the social and geopolitical background [23,24], the French and English Canadian culture [25], as well as the stance on climate change [26] apparent in Canada represent a particular context for the dissemination of climate change–related misinformation.

### Review Objectives

The main objective of this review is to assess the current state of knowledge about misinformation regarding climate change and related environmental events disseminated through social media.

The subobjectives of this review are to (1) report on the themes, actors, and sources of misinformation concerning climate change and related environmental events disseminated on social media, (2) evaluate how this dissemination of misinformation has evolved over time, and (3) assess whether a particular context surrounding this dissemination of misinformation prevails in Canada.

Considering the breadth of topics covered by our research objectives and subobjectives, we opted to conduct a scoping review, which allows us to effectively map the concepts underlying an area of research as well as the main sources and types of data available [27].

## Methods

### Approach

This scoping review will follow the methodological approach developed by Arksey and O’Malley [27] and advanced by Levac et al [28]. This approach entails the following 5 steps: (1) identification of the research question; (2) identification of relevant studies; (3) selection of relevant and reliable studies; (4) charting the data from the included studies; and (5) collating, summarizing, and reporting the findings.

The PRISMA-ScR (Preferred Reporting Items for Systematic Reviews and Meta-Analyses extension for Scoping Reviews) checklist [29] and the best practice guidance and reporting items for the development of scoping review protocols [30] were also used to develop this protocol.

### Identification of the Research Questions

By conducting this exploratory review, we aim to answer the following research questions: (1) What is the current state of knowledge about misinformation related to climate change and related environmental events circulating on social media? More specifically, (2) What are the themes, actors, and sources of misinformation about climate change and related environmental events disseminated on social media? (3) How has the dissemination of this misinformation evolved over time? (4) Is there a context particular to Canada, especially given the French and English Canadian background, surrounding the spread of misinformation about climate change and related environmental events through social media?

### Identification of Relevant Studies

#### Bibliographic Database Search

The literature search strategy was developed iteratively in collaboration with a specialized librarian (EB). First, 4 bibliographic databases relevant to our review topics were selected: MEDLINE (Ovid), Embase (Ovid), Web of Science, and GreenFILE. Free-text keywords were then identified through the databases for each of the 3 concepts covered by our research questions, misinformation, climate change and related environmental events, and social media (Table 1). In addition, controlled vocabulary search terms (thesaurus terms used by electronic bibliographic databases) were specified, as used by the MEDLINE, Embase, and GreenFILE search tools (Table 2). Search strategies specific to each database were defined (Multimedia Appendix 1).

**Table 1:** Concept associated free-text keywords identified for databases searches.

Concept	Free-text keywords^a^ (MEDLINE, Embase, Web of Science, and GreenFILE)
Climate change and related environmental events	((climate OR climatic) AND (change*, warming, issue*, vulnerabilit*, emergenc*, action*, crisis, disaster*, variabilit*, science, scientist*, modification*, topic*)), global warming, earth warming, greenhouse effect*, greenhouse gas*, drought*, flood*, hot temperature*, deglaciation, desertification, natural disaster*, heat wave*, hurricane*, tornado*, typhoon*, wildfire*, wild fire*, storm*, cyclone*, sea level* rise, ((extreme) AND (cold, weather, heat, temperature)), temperature* rising
Misinformation	disinform*, misinform*, dis inform*, mis inform*, malinfor*, mal infor*, infodem*, infobesit*, rumor*, rumour*, hoax*, fallac*, conspirac*, myth*, gossip*, propaganda, skeptic*, sceptic*, infoxication, veracity, polariz*, polaris*, controvers*, denial*, dessent*, contest*, deny, denier*, ((inaccurate, false, fake, poor quality, low quality, misleading, distorted) AND (information*, news, communication*))
Social media	((social, digital, onlin*) AND (media*, information*, network )), ((cyber, electronic, internet, onlin*, virtual, web*) AND (chat*, communit*, communicat*, conversat*, discussion, forum*, group*, messag*, network*, posts, posted, posting, share, shared, sharing, social)), blog, web 2.0, web 2.0s, webcast, streaming, podcast*, facebook, youtube, twitter, X, instagram, linkedin, linked in, flickr, pinterest, tiktok or tik tok, whatsapp, snapchat, reddit, telegram, wechat, hashtag*, hash tag*, tweet*, myspace, facetime, vlog*, influencer*, media* expos*

^a^Asterisks indicates various terminations are included.

**Table 2:** Concept associated controlled vocabulary search terms identified for bibliographic databases searches.

Concept	Databases’ controlled vocabulary search terms	
	MEDLINE	Embase
Climate change and related environmental events	Climate change, greenhouse effect, greenhouse gases, droughts, floods, cyclonic storms, sea level rise, and extreme weather	Climate change, greenhouse effect, greenhouse gas, drought, flooding, storms, sea level rise, hurricanes, heat waves, and extreme weather
Misinformation	Misinformation, disinformation, and information dissemination	Misinformation, disinformation, and information dissemination
Social media	Social media, blogging, communications media, and online social networking	Social media, blogging, mass medium, and online social network

#### Gray Literature Search

To complement the bibliographic database searches, we also developed a gray literature search strategy in collaboration with a specialized librarian (EB). In applying this strategy, we will consult Google, the Overton database, as well as dissertations and theses repositories. Using free-text keywords identified for the bibliographic database search strategies (Table 1), we will search through Google for textbooks, books, dissertations, and theses or reports covering our 3 research concepts in a combined manner. The first 100 Google search results will be screened for relevant publications. We will also seek reports related to our research concepts throughout the Overton database, which gathers policy documentation produced by or for policy makers from over 188 countries. These documents derive from governmental sources (government agencies, federal and provincial institutions), intergovernmental organizations (United Nations, World Health Organization, Organisation for Economic Co-operation and Development, and the European Union), think tanks and nongovernmental organizations (foundations, institutes and professional associations). Directories of international and national (Canada) institutional repositories will be screened for relevant dissertations and theses released by universities and other research institutions.

Gray literature searches will be documented in a Microsoft Excel spreadsheet, which will tabulate the following information for each search: date of search, source searched, terms or expressions used in search, number of results found, number of results retained, and comments. The relevance of publications from gray literature will be assessed using a simplified checklist itemizing authority, accuracy, coverage, objectivity, date, and significance.

Reference lists of relevant publications from bibliographic databases or gray literature could also be examined for additional references.

### Selection of Relevant and Reliable Studies

First, publications identified by the bibliographic database and gray literature searches will be imported into the bibliographic management software EndNote (Clarivate). Then, they will be exported to the Covidence platform (Veritas Health Innovation) to remove duplicates and facilitate the selection of publications to include in our review. Selection will be based on our defined inclusion and exclusion criteria (Textbox 1). The selection of publications will be piloted on a subset of potentially relevant sources and thereafter carried out independently by 2 members of the research team. Disagreements between the 2 reviewers will be settled by consensus, consulting a third research team member if necessary. The publication type filters of the Embase (Ovid) and Web of Science databases will be used to extract relevant publications.

**Criteria for including or excluding publications in the review.**

Inclusion criteria

- Covers misinformation about climate change and related environmental events disseminated on social media
- Provides author’s name
- Provides publication date
- Published on or after January 1, 2000
- Available in full-text version
- Published in English or French
- Publication types: peer-reviewed original article, systematic literature review, meta-analysis, scoping literature review, textbook, book, dissertation, thesis, or report

Exclusion criteria

- Publication types: opinion piece, media article, written or video commentary, editorial, press release, speeches, letter to the editor, conference proceeding, or data paper

A first selection round, based on publication titles and available abstracts, will be carried out. Published work covering misinformation related to climate change and related environmental events disseminated by social media will be included in order to answer our research questions. Publications addressing misinformation about issues other than climate change and related environmental events or disseminated through sources other than social media will be excluded, as not specifically related to our research questions.

A second selection round, based on the full text of publications, will define which publications are ultimately to be assessed. Publications that do not provide the author’s name and publication date will be excluded as they do not provide all the information required for data extraction. Given that the first social media platforms emerged in the 2000s, publications issued before January 1, 2000, will not be considered. Material not available in full-text version or published only in languages other than English or French will be excluded due to our inability to fully access their content. Publications in the form of peer-reviewed original articles, systematic literature reviews, meta-analyses, and scoping literature reviews will be included as the review process ensures the validity of these types of evidence-based publications. Textbooks, books, dissertations, theses, and reports will also be included as they are also typically reviewed. Quantitative, qualitative, and mixed methods studies will be considered to take into account the various aspects of our research concepts. Primary sources will be excluded if already incorporated into an included evidence synthesis unless the data they contain are not reported in the evidence synthesis. Opinion pieces, media articles, written or video commentaries, editorials, press releases, speeches, letters to the editor, conference proceedings and data papers will not be considered as they may not have been reviewed or be evidence-based.

The number of publications selected at each stage will be reported in a PRISMA (Preferred Reporting Items for Systematic reviews and Meta-Analyses) flow diagram [31]. Excluded publications and the reasons for exclusion at the full-text round of screening will be reported in the final review.

Considering the broad nature of scoping reviews, it is possible that research, screening, and selection may lead to the identification of new research terms or concepts, or new sources of data [30]. The methodology may need to be adapted accordingly. In that eventuality, any adjustments made will be noted, justified, and reported in the final review.

### Charting the Data From the Included Studies

Data relevant to answering our research questions will be extracted from the selected publications. An Excel data extraction form will be used to chart the following main items from each publication: title of publication, year of publication, themes, actors and sources of climate change-related misinformation reported, and conclusion (key messages). Also, extracting information on the geographic area referenced will make it possible to determine whether the publication reports on misinformation in a Canadian context. As there are multiple frameworks for defining and evaluating misinformation, the frameworks used in publications to classify information as misinformation will also be charted. As the extraction form is being iteratively developed specifically for this review, a preliminary version, which is subject to further adjustments, is presented here (Textbox 2). As recommended by Pollock et al [32], a pilot test of the extraction form will be conducted independently by each of the 2 reviewers for 10 of each type of publication included in the review to ensure that the form is accurate and in order to make any required adjustments. Inconsistencies revealed by testing will be discussed and resolved by consensus among the 2 reviewers, consulting a third research team member whenever required. Adjustments made to the extraction form and rationales will be noted and reported in the final review.

Using the tested standardized extraction form, data extraction from each included publication will be performed by a first reviewer. Extraction from each publication included will be reviewed by a second reviewer to validate accuracy and completeness [32]. If the extraction form needs to be updated in an iterative process, these revisions and rationales will also be noted and reported. Discrepancies occurring during data extraction will be discussed and settled by consensus between the 2 reviewers, consulting a third research team member if necessary. Authors of publications may be contacted to obtain and confirm data if needed.

The final version of the extraction form and the list and definitions of items extracted from publications will be presented in the final review. Any data assumptions and simplifications made will also be noted and presented in the final review.

**Preliminary data extraction form.**

- Title of publication:
- Type of publication (original article, review, book, thesis, report, etc):
- Author’s name:
- Year of publication:
- Geographical area:
- In the case of a study:
  Aim of study:
  Study population:
  Method:
  Results:
- Climate change–related topics (global warming, greenhouse effect, extreme weather events, natural disasters, etc) covered:
- Social media platforms covered:
- Framework used to define and evaluate misinformation:
- Themes of climate change–related misinformation reported:
- Actors tied to climate change–related misinformation reported:
- Sources of climate change–related misinformation reported:
- Conclusion (key messages):
- Other:

### Collating, Summarizing, and Reporting the Findings

An overview and description of all the included publications and extracted data will be presented in the final review. For each publication, the citations, characteristics, and relevant data will be reported in tabular format.

Charted data will be processed by the research team in order to answer the general and specific research questions. To summarize findings, we will use qualitative methods to regroup data and identify key themes relevant to our research question and generate meaningful insights. The results will be presented using narrative format or visual representations such as maps, diagrams, or tables, ensuring that key findings are reported in a clear and concise manner.

## Results

Funding for this project was provided in December 2023. The first step in the methodological approach, the identification of the research questions, was achieved by the research team in January 2024. Accordingly, the literature search strategies for the bibliographic databases were iteratively developed from January to March 2024 for MEDLINE, Embase, and Web of Science by EB and VT and in July 2024 for GreenFILE by EB. The gray literature search strategy was developed in July 2024 by EB and VT.

The MEDLINE, Embase, and Web of Science databases were searched according to final strategies on March 26, 2024, by VT. The first round of selection of publications identified through these databases was achieved in April 2024 by MV and VT. As of July 2024, the second round of selection was being pursued by MV and VT. The search through the GreenFILE database and the gray literature is scheduled for October to December 2024.

The final selection of publications, the charting, collating, and summarizing of data, as well as the reporting of findings is planned for 2025. We intend to publish the findings of this scoping review as a research paper in a dedicated peer-reviewed journal.

## Discussion

### Expected Outcomes

This protocol will enable us to assess the current state of knowledge about misinformation regarding climate change and related environmental events on social media from an updated perspective, including the latest social media platforms that could not have been covered by earlier reviews [9,21]. This will also enable us to answer our more specific research questions by identifying the themes, actors, and sources of misinformation concerning climate change and related environmental events disseminated on social media and revealing how these trends have changed over time.

Trends could also be identified by collating and summarizing data on climate change and related environmental events according to topics (global warming, greenhouse effect, extreme weather events, natural disasters, etc), time periods, social media platforms, and geographical areas covered by the selected publications. Identifying misinformation trends and variations over time and between platforms will help guide interventions aimed at raising awareness and combating misinformation. For example, findings emerging from this review could steer the development of programs teaching prevention, detection, or correction of the various found forms of misinformation about climate change and related environmental events found circulating recently on specific social media platforms. Findings from this review could also potentially add to knowledge and support further research about other related topics, such as people’s perceptions [33,34], mental health conditions [35,36], and behaviors [37-39] linked to climate change. The findings could also produce insights about echo chambers and misinformation networks leading to a better understanding of the argumentation, values, cultural contexts, and beliefs behind the spread of misinformation about climate change and related environmental events on social media. As climate change–related misinformation also circulates offline [7], this review will support a broader understanding of these issues by enriching knowledge through the addition of updated insights about such circulation on social media.

As misinformation is known to undermine actions and support in the fight against climate change [3,6], the potential identification of a particularly Canadian context of themes, actors and sources of climate change-related misinformation on social media could help point toward avenues for future research and guide the planning and implementation of local actions aimed at addressing this issue [9,39,40]. Finally, this scoping review may lead us to report on gaps in the current state of knowledge.

### Limitations

This protocol for a scoping review has a few limitations. To streamline our queries among the selected databases, the social media search strategies include keywords specific to platforms targeting only occidental populations and offering content in English or French. Studies on climate change misinformation circulating on other social media, such as the Chinese platform Weibo (Sina Corp) [41], could, however, be identified by our selection of keywords referring to social media in broad terms. Since this scoping review aims to map the overall landscape of research on climate change and related environmental misinformation on social media, study approaches and definitions of misinformation used by the different publications included may vary widely. This could make it difficult to extract data, collate, summarize, and collectively report the findings from the different publications. Following defined data extraction and analysis processes, entailing the use of a detailed standardized data extraction form and spreadsheets, will facilitate the synthesis of findings.

### Conclusions

By developing this scoping review protocol, we aim to facilitate our review of the current state of knowledge on misinformation regarding climate change and related environmental events on social media and, more specifically, to identify the themes, actors, and sources of such misinformation. Conducting the review would also allow us to identify a local context for this issue. By enhancing this knowledge base in an updated and contextualized manner, we intend to facilitate the targeting of efforts to combat misinformation related to climate change disseminated on social media.

## Appendix

Multimedia Appendix 1 Databases search strategies.

Multimedia Appendix 2 Examples of prompts similar to those used for proofreading.

## Abbreviations

PRISMA: Preferred Reporting Items for Systematic Reviews and Meta-Analyses
PRISMA-ScR: Preferred Reporting Items for Systematic reviews and Meta-Analyses extension for Scoping Reviews

## Data Availability

Data sharing is not applicable to this article as no data sets were generated or analyzed during the development of this study protocol.
