# Supplementary material for: Misinformation About Climate Change and Related Environmental Events on Social Media: Protocol for a Scoping Review
Source: JMIR Res Protoc. 2024 Oct 31;13:e59345. doi: 10.2196/59345 (PMC11565082; doi:10.2196/59345)
Supplement: Multimedia Appendix 1 [file resprot_v13i1e59345_app1.pdf]

Multimedia Appendix 1

Databases search strategies

Query #	MEDLINE (Ovid) syntax<br>(final version finalized and launched in databases on March 26, 2024)
**1**	
	(((climate or climatic) adj3 (change* or warming or issue* or vulnerabilit* or emergenc* or action* or crisis or disaster* or variabilit* or science or scientist* or modification* or topic*)) or global warming or (earth adj2 warming) or greenhouse effect* or greenhouse gas* or drought* or flood* or hot temperature* or deglaciation or desertification or natural disaster* or heat wave* or hurricane* or tornado* or typhoon* or wildfire* or wild fire* or storm* or cyclone* or sea level* rise or (extreme adj2 (cold or weather or heat or temperature*)) or (temperature* adj1 rising)).ti,ab,kw
**2**	
	exp climate change/ or greenhouse gases/ or greenhouse effect/ or droughts/ or floods/ or cyclonic Storms/ or sea level rise/ or exp extreme weather/
**3**	
	1 or 2
**4**	
	(((social or digital or onlin*) adj2 (media* or information* or network)) or ((cyber or electronic or internet or onlin* or virtual or web*) adj3 (chat* or communit* or communicat* or conversat* or discussion or forum* or group* or messag* or network* or posts or posted or posting or share or shared or sharing or social)) or blog or "web 2.0" or "web 2.0s" or webcast or streaming or podcast* or facebook or youtube or twitter or X or instagram or linkedin or linked in or flickr or pinterest or tiktok or tik tok or whatsapp or snapchat or reddit or telegram or wechat or hashtag* or hash tag* or tweet* or myspace or facetime or vlog* or influencer* or (media* adj2 expos*)).ti,ab,kw
**5**	
	social media/ or blogging/ or communications media/ or online social networking/
**6**	
	4 or 5
**7**	
	(disinform* or misinform* or dis inform* or mis inform* or malinfor* or mal infor* or infodem* or infobesit* or rumo?r * or hoax* or fallac* or conspirac* or myth or myths or gossip* or propaganda or skeptic* or sceptic* or infoxication or veracity or polariz* or polaris* or controvers* or denial* or dessent* or contest* or deny or denier* or ((inaccurate or false or fake or poor quality or low quality or misleading or distorted) adj3 (information* or news or communication*))).ti,ab,kw

**8**	
	disinformation/ or exp information dissemination/
**9**	
	7 or 8
**10**	
	3 and 6 and 9
**11**	
	limit 10 to yr="2000-Current"

Query #	Embase (Ovid) syntax<br>(final version finalized and launched in databases on March 26, 2024)
**1**	
	(((climate or climatic) adj3 (change* or warming or issue* or vulnerabilit* or emergenc* or action* or crisis or disaster* or variabilit* or science or scientist* or modification* or topic*)) or global warming or (earth adj2 warming) or greenhouse effect* or greenhouse gas* or drought* or flood* or hot temperature* or deglaciation or desertification or natural disaster* or heat wave* or hurricane* or tornado* or typhoon* or wildfire* or wild fire* or storm* or cyclone* or sea level* rise or (extreme adj2 (cold or weather or heat or temperature*)) or (temperature* adj1 rising)).ti,ab,kw
**2**	
	exp climate change/ or greenhouse effect/ or greenhouse gas/ or drought/ or flooding/ or hurricane/ or sea level rise/ or exp heat wave/ or storm/ or exp extreme weather/
**3**	
	1 or 2
**4**	
	(((social or digital or onlin*) adj2 (media* or information* or network*)) or ((cyber or electronic or internet or onlin* or virtual or web*) adj3 (chat* or communit* or communicat* or conversat* or discussion or forum* or group* or messag* or network* or posts or posted or posting or share or shared or sharing or social)) or blog* or "web 2.0" or "web 2.0s" or webcast or streaming or podcast* or facebook or youtube or twitter or X or instagram or linkedin or linked in or flickr or pinterest or tiktok or tik tok or whatsapp or snapchat or reddit or telegram or wechat or hashtag* or hash tag* or tweet* or myspace or facetime or vlog* or influencer* or (media* adj2 expos*)).ti,ab,kw
**5**	
	social media/ or exp blogging/ or mass medium/ or online social network/
**6**	
	4 or 5
**7**	
	(disinform* or misinform*or dis inform* or mis inform* or malinfor* or mal infor* or infodem* or infobesit* or rumo?r* or hoax* or fallac* or conspirac* or myth or myths or gossip* or propaganda or skeptic* or sceptic* or infoxication or veracity or polariz* or polaris* or controvers* or denial* or dessent* or contest* or deny or denier* or ((inaccurate or false or fake or poor quality or low quality or misleading or distorted) adj3 (information* or news or communication*))).ti,ab,kw
**8**	
	exp misinformation/ or exp information dissemination/
**9**	

	7 or 8
**10**	
	3 and 6 and 9
**11**	
	limit 10 to yr="2000 -Current"

Query #	Web of Science syntax<br>(final version finalized and launched in databases on March 26, 2024)
**1**	
	TI=((((climate or climatic) NEAR/3 (change* or warming or issue* or vulnerabilit* or emergenc* or action* or crisis or disaster* or variabilit* or science or scientist* or modification* or topic*)) or global warming or (earth NEAR/2 warming) or "greenhouse effect*" or "greenhouse gas*" or drought* or flood* or "hot temperature*" or deglaciation or desertification or "natural disaster*" or "heat wave*" or hurricane* or tornado* or typhoon* or wildfire* or "wild fire*" or storm* or cyclone* or "sea level* rise" or (extreme NEAR/2 (cold or weather or heat or temperature*)) or (temperature* NEAR/1 rising)))
**2**	
	AB=((((climate or climatic) NEAR/3 (change* or warming or issue* or vulnerabilit* or emergenc* or action* or crisis or disaster* or variabilit* or science or scientist* or modification* or topic*)) or global warming or (earth NEAR/2 warming) or "greenhouse effect*" or "greenhouse gas*" or drought* or flood* or "hot temperature*" or deglaciation or desertification or "natural disaster*" or "heat wave*" or hurricane* or tornado* or typhoon* or wildfire* or "wild fire*" or storm* or cyclone* or "sea level* rise" or (extreme NEAR/2 (cold or weather or heat or temperature*)) or (temperature* NEAR/1 rising)))
**3**	
	AK=((((climate or climatic) NEAR/3 (change* or warming or issue* or vulnerabilit* or emergenc* or action* or crisis or disaster* or variabilit* or science or scientist* or modification* or topic*)) or global warming or (earth NEAR/2 warming) or "greenhouse effect*" or "greenhouse gas*" or drought* or flood* or "hot temperature*" or deglaciation or desertification or "natural disaster*" or "heat wave*" or hurricane* or tornado* or typhoon* or wildfire* or "wild fire*" or storm* or cyclone* or "sea level* rise" or (extreme NEAR/2 (cold or weather or heat or temperature*)) or (temperature* NEAR/1 rising)))
**4**	
	KP=((((climate or climatic) NEAR/3 (change* or warming or issue* or vulnerabilit* or emergenc* or action* or crisis or disaster* or variabilit* or science or scientist* or modification* or topic*)) or global warming or (earth NEAR/2 warming) or "greenhouse effect*" or "greenhouse gas*" or drought* or flood* or "hot temperature*" or deglaciation or desertification or "natural disaster*" or "heat wave*" or hurricane* or tornado* or typhoon* or wildfire* or "wild fire*" or storm* or cyclone* or "sea level* rise" or (extreme NEAR/2 (cold or weather or heat or temperature*)) or (temperature* NEAR/1 rising)))
**5**	
	1 or 2 or 3 or 4
**6**	

	TI=((((social or digital or onlin*) NEAR/2 (media* or information* or network*)) or ((cyber or electronic or internet or onlin* or virtual or web*) NEAR/3 (chat* or communit* or communicat* or conversat* or discussion or forum* or group* or messag* or network* or posts or posted or posting or share or shared or sharing or social)) or blog* or "web 2.0" or "web 2.0s" or webcast or streaming or podcast* or facebook or youtube or twitter or X or instagram or linkedin or "linked in" or flickr or pinterest or tiktok or "tik tok" or whatsapp or snapchat or reddit or telegram or wechat or hashtag* or "hash tag*" or tweet* or myspace or facetime or vlog* or influencer* or (media* NEAR/2 expos*)))
**7**	
	AB=((((social or digital or onlin*) NEAR/2 (media* or information* or network*)) or ((cyber or electronic or internet or onlin* or virtual or web*) NEAR/3 (chat* or communit* or communicat* or conversat* or discussion or forum* or group* or messag* or network* or posts or posted or posting or share or shared or sharing or social)) or blog* or "web 2.0" or "web 2.0s" or webcast or streaming or podcast* or facebook or youtube or twitter or X or instagram or linkedin or "linked in" or flickr or pinterest or tiktok or "tik tok" or whatsapp or snapchat or reddit or telegram or wechat or hashtag* or "hash tag*" or tweet* or myspace or facetime or vlog* or influencer* or (media* NEAR/2 expos*)))
**8**	
	AK=((((social or digital or onlin*) NEAR/2 (media* or information* or network*)) or ((cyber or electronic or internet or onlin* or virtual or web*) NEAR/3 (chat* or communit* or communicat* or conversat* or discussion or forum* or group* or messag* or network* or posts or posted or posting or share or shared or sharing or social)) or blog* or "web 2.0" or "web 2.0s" or webcast or streaming or podcast* or facebook or youtube or twitter or X or instagram or linkedin or "linked in" or flickr or pinterest or tiktok or "tik tok" or whatsapp or snapchat or reddit or telegram or wechat or hashtag* or "hash tag*" or tweet* or myspace or facetime or vlog* or influencer* or (media* NEAR/2 expos*)))
**9**	
	KP=((((social or digital or onlin*) NEAR/2 (media* or information* or network*)) or ((cyber or electronic or internet or onlin* or virtual or web*) NEAR/3 (chat* or communit* or communicat* or conversat* or discussion or forum* or group* or messag* or network* or posts or posted or posting or share or shared or sharing or social)) or blog* or "web 2.0" or "web 2.0s" or webcast or streaming or podcast* or facebook or youtube or twitter or X or instagram or linkedin or "linked in" or flickr or pinterest or tiktok or "tik tok" or whatsapp or snapchat or reddit or telegram or wechat or hashtag* or "hash tag*" or tweet* or myspace or facetime or vlog* or influencer* or (media* NEAR/2 expos*)))
**10**	

	6 or 7 or 8 or 9
**11**	
	TI=((disinform* or misinform* or "dis inform*" or "mis inform*" or malinfor* or "mal infor*" or infodem* or infobesit* or rumo?r* or hoax* or fallac* or conspirac* or myth or myths or gossip* or propaganda or skeptic* or sceptic* or infoxication or veracity or polariz* or polaris* or controvers* or denial* or dessent* or contest* or deny or denier* or ((inaccurate or false or fake or "poor quality" or "low quality" or misleading or distorted) NEAR/3 (information* or news or communication*))))
**12**	
	AB=((disinform* or misinform* or "dis inform*" or "mis inform*" or malinfor* or "mal infor*" or infodem* or infobesit* or rumo?r* or hoax* or fallac* or conspirac* or myth or myths or gossip* or propaganda or skeptic* or sceptic* or infoxication or veracity or polariz* or polaris* or controvers* or denial* or dessent* or contest* or deny or denier* or ((inaccurate or false or fake or "poor quality" or "low quality" or misleading or distorted) NEAR/3 (information* or news or communication*))))
**13**	
	AK=((disinform* or misinform* or "dis inform*" or "mis inform*" or malinfor* or "mal infor*" or infodem* or infobesit* or rumo?r* or hoax* or fallac* or conspirac* or myth or myths or gossip* or propaganda or skeptic* or sceptic* or infoxication or veracity or polariz* or polaris* or controvers* or denial* or dessent* or contest* or deny or denier* or ((inaccurate or false or fake or "poor quality" or "low quality" or misleading or distorted) NEAR/3 (information* or news or communication*))))
**14**	
	KW=((disinform* or misinform* or "dis inform*" or "mis inform*" or malinfor* or "mal infor*" or infodem* or infobesit* or rumo?r* or hoax* or fallac* or conspirac* or myth or myths or gossip* or propaganda or skeptic* or sceptic* or infoxication or veracity or polariz* or polaris* or controvers* or denial* or dessent* or contest* or deny or denier* or ((inaccurate or false or fake or "poor quality" or "low quality" or misleading or distorted) NEAR/3 (information* or news or communication*))))
**15**	
	11 or 12 or 13 or 14
**16**	
	5 and 10 and 15

Query #	GreenFILE syntax<br>(final version finalized on July 29, 2024)
**1**	
	((**TI** (((climate or climatic) N3 (change* or warming or issue* or vulnerabilit* or emergenc* or action* or crisis or disaster* or variabilit* or science or scientist* or modification* or topic*)) or global warming or (earth N2 warming) or greenhouse effect* or greenhouse gas* or drought* or flood* or hot temperature* or deglaciation or desertification or natural disaster* or heat wave* or hurricane* or tornado* or typhoon* or wildfire* or wild fire* or storm* or cyclone* or sea level* rise or (extreme N2 (cold or weather or heat or temperature*)) or (temperature* N1 rising)) **OR AB**(((climate or climatic) N3 (change* or warming or issue* or vulnerabilit* or emergenc* or action* or crisis or disaster* or variabilit* or science or scientist* or modification* or topic*)) or global warming or (earth N2 warming) or greenhouse effect* or greenhouse gas* or drought* or flood* or hot temperature* or deglaciation or desertification or natural disaster* or heat wave* or hurricane* or tornado* or typhoon* or wildfire* or wild fire* or storm* or cyclone* or sea level* rise or (extreme N2 (cold or weather or heat or temperature*)) or (temperature* N1 rising))) **OR (DE** ("CLIMATE change" OR "ABRUPT climate change" OR "CLIMATE apocalypse" OR "GLOBAL temperature changes" OR "WEATHER & climate change" OR "global warming" OR "Greenhouse effect" OR "droughts" OR "floods" OR "natural disasters" OR "GLACIAL melting" OR "DESERTIFICATION" OR "HEAT waves (Meteorology)" OR "STORMS" OR "CYCLONES" OR "HURRICANES" OR "TORNADOES" OR "WILDFIRES" OR "ABSOLUTE sea level change" OR "EXTREME weather"))) **AND** ((**TI** (((social or digital or onlin*) N2 (media* or information* or network)) or ((cyber or electronic or internet or onlin* or virtual or web*) N3 (chat* or communit* or communicat* or conversat* or discussion or forum* or group* or messag* or network* or posts or posted or posting or share or shared or sharing or social)) or blog or "web 2.0" or "web 2.0s" or webcast or streaming or podcast* or facebook or youtube or twitter or X or instagram or linkedin or linked in or flickr or pinterest or tiktok or tik tok or whatsapp or snapchat or reddit or telegram or wechat or hashtag* or hash tag* or tweet* or myspace or facetime or vlog* or influencer* or (media* N2 expos*)) **OR AB** (((social or digital or onlin*) N2 (media* or information* or network)) or ((cyber or electronic or internet or onlin* or virtual or web*) N3 (chat* or communit* or communicat* or conversat* or discussion or forum* or group* or messag* or network* or posts or posted or posting or share or shared or sharing or social)) or blog or "web 2.0" or "web 2.0s" or webcast or streaming or podcast* or facebook or youtube or twitter or X or instagram or linkedin or linked in or flickr or pinterest or tiktok or tik tok or whatsapp or snapchat or reddit or telegram or wechat or

	hashtag* or hash tag* or tweet* or myspace or facetime or vlog* or influencer* or (media* N2 expos*)) **OR (DE** ("Social media" or "Virtual communities" or "Social networks" or "Microblogs" or "Websites" or "Digital communications"))) **AND ((TI** (disinform* or misinform* or dis inform* or mis inform* or malinfor* or mal infor* or infodem* or infobesit* or rumo?r * or hoax* or fallac* or conspirac* or myth or myths or gossip* or propaganda or skeptic* or sceptic* or infoxication or veracity or polariz* or polaris* or controvers* or denial* or dessent* or contest* or deny or denier* or ((inaccurate or false or fake or poor quality or low quality or misleading or distorted) N3 (information* or news or communication*))) **OR AB** (disinform* or misinform* or dis inform* or mis inform* or malinfor* or mal infor* or infodem* or infobesit* or rumo?r * or hoax* or fallac* or conspirac* or myth or myths or gossip* or propaganda or skeptic* or sceptic* or infoxication or veracity or polariz* or polaris* or controvers* or denial* or dessent* or contest* or deny or denier* or ((inaccurate or false or fake or poor quality or low quality or misleading or distorted) N3 (information* or news or communication*)))) **OR (DE**("DISINFORMATION" or "Conspiracy theories"))
**2**	
	**DE**"CLIMATE change skepticism" **AND** ((**TI** (((social or digital or onlin*) N2 (media* or information* or network)) or ((cyber or electronic or internet or onlin* or virtual or web*) N3 (chat* or communit* or communicat* or conversat* or discussion or forum* or group* or messag* or network* or posts or posted or posting or share or shared or sharing or social)) or blog or "web 2.0" or "web 2.0s" or webcast or streaming or podcast* or facebook or youtube or twitter or X or instagram or linkedin or linked in or flickr or pinterest or tiktok or tik tok or whatsapp or snapchat or reddit or telegram or wechat or hashtag* or hash tag* or tweet* or myspace or facetime or vlog* or influencer* or (media* N2 expos*)) **OR AB** (((social or digital or onlin*) N2 (media* or information* or network)) or ((cyber or electronic or internet or onlin* or virtual or web*) N3 (chat* or communit* or communicat* or conversat* or discussion or forum* or group* or messag* or network* or posts or posted or posting or share or shared or sharing or social)) or blog or "web 2.0" or "web 2.0s" or webcast or streaming or podcast* or facebook or youtube or twitter or X or instagram or linkedin or linked in or flickr or pinterest or tiktok or tik tok or whatsapp or snapchat or reddit or telegram or wechat or hashtag* or hash tag* or tweet* or myspace or facetime or vlog* or influencer* or (media* N2 expos*)) OR (DE ("Social media" or "Virtual communities" or "Social networks" or "Microblogs" or "Websites" or "Digital communications")))
**3**	
	**1 OR 2**
