# Supplementary material for: Misinformation About Climate Change and Related Environmental Events on Social Media: Protocol for a Scoping Review
Source: JMIR Res Protoc. 2024 Oct 31;13:e59345. doi: 10.2196/59345 (PMC11565082; doi:10.2196/59345)
Supplement: Multimedia Appendix 2 [file resprot_v13i1e59345_app2.pdf]

Example of prompts used for proofing and editing some sentences of the manuscript were:

Check this sentence for grammar and syntax?

Make this sentence clearer and more concise?

Revise this sentence to sound more formal and suitable for a scientific manuscript:
